# One Heat Shock Transcription Factor Confers High Thermal Tolerance in Clematis Plants

**DOI:** 10.3390/ijms22062900

**Published:** 2021-03-12

**Authors:** Rui Wang, Chanjuan Mao, Changhua Jiang, Long Zhang, Siyuan Peng, Yi Zhang, Shucheng Feng, Feng Ming

**Affiliations:** 1 Shanghai Key Laboratory of Plant Molecular Sciences, College of Life Sciences, Shanghai Normal University, Shanghai 200234, China; wrwangrui2020@yeah.net (R.W.); cjmao@shnu.edu.cn (C.M.); zlong791@163.com (L.Z.); m18201923085@163.com (S.P.); yunx8973@163.com (Y.Z.); 2 The Biotechnology Research Institute, Shanghai Academy of Agricultural Sciences, Shanghai 201106, China; 3 Shanghai Botanical Garden, Shanghai Urban Plant Resources Development and Application Engineering Technology Research Center, Shanghai 200231, China; 051023046@fudan.edu.cn

**Keywords:** *Clematis*, heat stress, transcriptome analysis, *CvHSF30-2*, *CvHSFB2a*, VIGS

## Abstract

Clematis plants play an important role in botanical gardens. Heat stress can destroy the activity, state and conformation of plant proteins, and its regulatory pathway has been well characterized in *Arabidopsis* and some crop plants. However, the heat resistance response mechanism in horticultural plants including *Clematis* has rarely been reported. Here, we identified a heat-tolerant clematis species, *Clematis vitalba*. The relative water loss and electrolytic leakage were significantly lower under heat treatment in *Clematis vitalba* compared to Stolwijk Gold. Differential expression heat-tolerant genes (HTGs) were identified based on nonparametric transcriptome analysis. For validation, one heat shock transcription factor, *CvHSF30-2*, extremely induced by heat stimuli in *Clematis vitalba*, was identified to confer tolerance to heat stress in *Escherichia coli* and *Saccharomyces cerevisiae*. Furthermore, silencing of *HSF30-2* by virus-induced gene silencing (VIGS) led to heat sensitivity in tobacco and *Clematis*, suggesting that the candidate heat-resistant genes identified in this RNA-seq analysis are credible and offer significant utility. We also found that *CvHSF30-2* improved heat tolerance of *Clematis vitalba* by elevating heat shock protein (HSP) expression, which was negatively regulated by *CvHSFB2a*. Taken together, this study provides insights into the mechanism of *Clematis* heat tolerance and the findings can be potentially applied in horticultural plants to improve economic efficiency through genetic approaches.

## 1. Introduction

*Clematis*, also known as the clematis peony, is a genus of about 355 species of buttercup, mainly distributed in the temperate regions north of the Earth’s equator, which are typical woody vines. *Clematis* plants play an important role in botanical gardens due to their unique flowers, rich colors and wire-like stems and are praised as the “Queen of Vines” [1]. *Clematis* can be seen in most botanical gardens, parks and family gardens. Additionally, *Clematis* is a plant source of many medicinal active ingredients and its specialized metabolites can be used as medicine to disperse wind damp, unclog channels and ease pain [2].

Thermal stress is a major threat to the cell, causing protein denaturation and compromising membrane integrity, leading to the death of plant cells [3,4]. Understanding the balance between temperature perception and plant defense can provide a foundation for enhancing plant tolerance (defined as the ability to compensate in part for fitness decrements caused by stress [5]) to thermal stress and improving economic efficiency. Eukaryotic cells respond to heat shock by elevating the transcription of heat shock proteins (HSPs), many of which function in preventing or repairing the damage caused by heat stress and thus confer enhanced thermotolerance [3,6]. Recent work has revealed that plant HSPs are classified into five major groups: HSP100s, HSP90s, HSP70s, HSP60s and small HSPs (sHSPs) [7,8]. *Arabidopsis* cytosolic HSP90 regulates the heat shock response and is responsible for heat acclimation [9]. The chloroplast small heat shock protein HSP21 is essential for the development of chloroplasts during heat stress [10]. The sHsp26 family protects the photosynthetic machinery during episodes of high-temperature stress in wheat [11].

HSPs are transcriptionally induced by heat shock factor (HSF)-class transcription factors (TFs) upon activation by heat stress [6,12]. The HsfA1 group is considered as the master regulator of heat stress response (HSR) [13]. Genetic evidence shows that HsfA1a and HsfA1b are involved in the induction of a number of Hsp genes in the early phase of HSR in *Arabidopsis* [14]. In tomato (*Lycopersicon esculentum*), HsfA1a plays an important role in regulating HSR [15]. HsfA2 is highly and exclusively expressed under heat stress and becomes the dominant HSF under long-term heat stress (HS) [13]. Excessive expression of *Arabidopsis* HsfA2 was found to improve heat tolerance in an hsfA1a,b,d,e mutant [16]. Increased thermotolerance was also obtained by ectopic expression of rice HsfA2a and wheat HsfA2d in *Arabidopsis* [17,18]. Some HSF proteins have been reported to be involved in HsfA1-independent pathways under heat shock [19]. A rice (*Oryza sativa*) mutant, *spl7*, which is sensitive to elevated temperature, has been shown to have a missense mutation in an *Hsf* gene belonging to the HsfA4 group [20]. It has also been reported that HSFB TFs, e.g., *Arabidopsis* HsfB1 and HsfB2b, function as repressors in the expression of *HsfA* and several heat shock proteins [21]. Unlike that in *Arabidopsis* and some crop plants, the heat resistance response mechanism in horticultural plants including *Clematis* has been scarcely reported on.

Virus-induced gene silencing (VIGS) is a burgeoning and useful technology that is based on the plant autoimmune system. When plants face viral infection, the endogenous gene homologous to the insert fragment in the VIGS vector can be degraded by post-transcriptional gene silencing (PTGS) [22,23]. Some plant viruses have been used to develop VIGS vectors; e.g., the tobacco rattle virus (TRV) [24], tobacco mosaic virus (TMV) [25], tomato golden mosaic virus (TGMV) [26] and potato virus X (PVX) [27]. VIGS is an effective reverse-genetics tool for the gene function study of plants [28]. Knockdown of endogenous F3′H in *Phalaenopsis* via VIGS to assess whether delphinidin could accumulate has been conducted previously [29], and in another study, *RcAP1* was silenced by using VIGS in Rose “Old Blush” to verify its function in flower bud formation [30].

In this study, one heat-tolerant *Clematis* species, *Clematis vitalba*, was identified based on physiological indexes. Transcriptome analysis showed that HSF TFs are essential for thermotolerance in *Clematis vitalba*. For validation, one heat-shock transcription factor, *CvHSF30-2*, extremely induced by heat stimuli in *Clematis vitalba*, was identified as conferring resistance to a species of stresses to *Escherichia coli* and *Saccharomyces cerevisiae*. The effects of *CvHSF30-2* on heat stress in tobacco were verified by the VIGS technique. In addition, we established the method of *Clematis* VIGS to provide a theoretical basis for the study of its gene function. We also found that *CvHSF30-2* improved the heat tolerance of *Clematis vitalba* by elevating *HSP* expression, which was negatively regulated by *CvHSFB2a*. Our findings uncover the thermotolerance mechanism in *Clematis* varieties and have potential applications in agriculture in improving the economic efficiency of horticultural plants through genetic approaches.

## 2. Results

### 2.1. Heat Shock Phenotypes of Different Clematis Species

**Figure 1:**
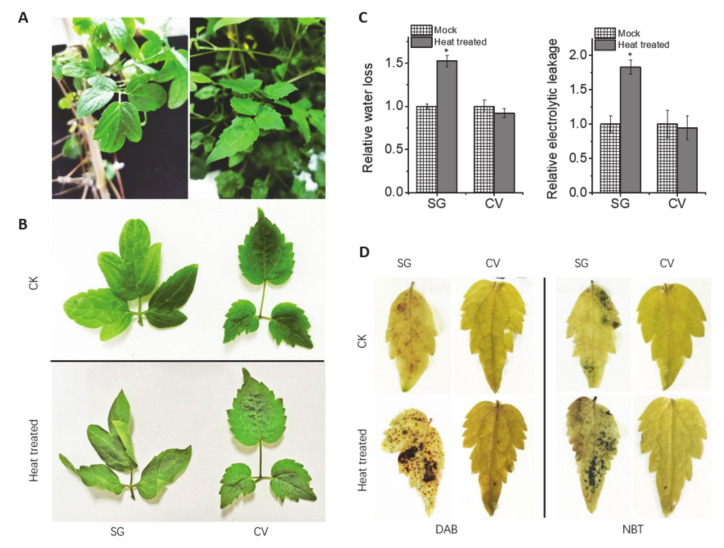
Two *Clematis* varieties and physiological changes after high-temperature treatment. (**A**) Two *Clematis* plants: *Clematis vitalba* (Cv, left) and *Clematis* “Stolwijk Gold” (SG, right). (**B**) Phenotype of the two *Clematis* plants after heat treatment. (**C**) Change of relative water loss and relative electrolytic leakage after high-temperature treatment (42 °C for 2 h) in *Clematis* varieties. (**D**) Diaminobenzidine (DAB) and nitro blue tetrazolium (NBT) staining of the two *Clematis* varieties’ leaves after high-temperature treatment (42 °C for 2 h).

*Clematis vitalba* (Cv) is an original species of *Clematis* with branched, grooved stems, deciduous leaves and scented green-white flowers with fluffy underlying sepals. Due to its disseminatory reproductive system, vitality and climbing behavior, *Clematis vitalba* is an invasive plant in many places, such as New Zealand (http://www.iucngisd.org/gisd/species.php?sc=157 (accessed on 3 May 2020)). *Clematis alpina* “Stolwijk Gold” (SG) belongs to the ornamental cultivars of *Clematis* (Figure 1A), as recognized by the Royal Horticultural Society of England (https://www.rhs.org.uk/Plants/236939/Clematis-alpina-Stolwijk-Gold-(A)/Details (accessed on 3 May 2020)).

In order to identify heat tolerance differences, Cv and SG were treated with 42 °C for 2 h. It can be seen that the leaves of SG were largely wilting, while those of Cv were totally normal (Figure 1B), suggesting that Cv is one species with high thermotolerance. In addition, relative water loss and electrolytic leakage significantly increased in SG, whereas Cv did not show much difference from the control (Figure 1C), meaning it suffered less membrane damage under heat stress. Moreover, nitro blue tetrazolium (NBT) and diaminobenzidine (DAB) staining revealed that SG had high reactive oxygen species (ROS) accumulation (Figure 1D) after heat treatment, but ROS accumulation was almost unchanged in Cv, indicating that Cv had a very strong antioxidant system under high temperature. All these results illustrated that Cv is tolerant to high temperature while SG is sensitive to heat shock.

### 2.2. Identification of Heat-Tolerant Genes and Their Differential Expression in Clematis vitalba

**Figure 2:**
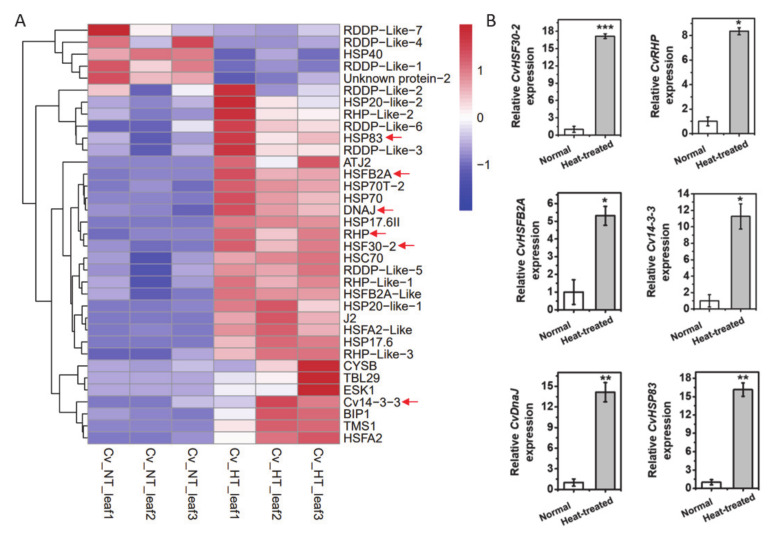
RNA-seq analysis of *Clematis vitalba* after high-temperature treatment. (**A**) Heat map of differentially expressing heat-related genes (HRGs). The genes chosen for the qPCR assay are marked by arrows. The descriptions of the names of HRGs are shown in Supplementary Table S2. (**B**) qPCR analysis of some HRGs. * *p* < 0.05; ** *p* < 0.01. *** *p* < 0.001.

To reveal the molecular mechanism of heat tolerance in Cv, an RNA-seq analysis was performed for leaves from Cv and SG kept at 22 °C (NT) or treated at 42 °C (HT) for 2 h, for which each treatment included three biological replicates. Principal components analysis (PCA) was performed to show the similarity of replicates (Figure S1A,B). Compared with the control, totals of 6506 and 6470 differently expressing genes (DEGs) were detected in heat-shocked Cv and SG, respectively (*p* < 0.05) (Supplementary Figure S1C). In Cv, 656 transcription factors were identified in the RNA-seq and 58 of them were differently expressed in response to heat shock, including NAC domain-containing protein 100, MADS-box protein AGL80, R2R3-myb transcription factor, transcription repressor MYB6-like, ethylene-responsive transcription factor ERF014 and heat shock factor (Supplementary Figure S1D; Supplementary Table S1). Furthermore, 35 heat-related genes (HRGs) identified by Gene Ontology (GO) annotation were detected in the Cv heat response, including heat shock transcription factors (HSFs), heat shock proteins, 14-3-3 proteins, ribonuclease H protein (RHP) and dnaJ protein (Figure 2A; Supplementary Table S2 shows the description of HRGs), which function in heat signal transduction, transcription regulation and protein homeostasis. In addition, we examined the expression of some genes participating in heat response with a qPCR assay (Figure 2B). The mRNA levels of *CvHSF30-2*, *CvHSFB2a*, *CvHSP83*, ribonuclease H protein (RHP), 14-3-3 proteins and dnaJ protein were significantly upregulated in Cv (Figure 2B), which was consistent with the RNA-seq results. Notably, hardly any heat-related genes overlapped between SG and CV after heat shock (Supplementary Figure S1C), indicating that the heat response mechanism of Cv differs from that of SG and that Cv might have its own special heat tolerant mechanism. It has been reported that transcriptional factors play vital roles in plant development and environmental response, among which HSFs have been reported to participate in plant thermotolerance [6]. Thus, we gave priority to the function Hsf in *Clematis* thermotolerance, although other transcription factors may also play a part in this process. Pertinent to our interest, *CvHSF30-2*, which intersected in differently expressing transcriptional factors and HRGs, showed great induction of heat shock. Thus, we speculate that *CvHSF30-2* may play an important part in the thermotolerance of Cv.

### 2.3. CvHSF30-2 Serves as a Typical Type-A Heat Shock Transcription Factor

**Figure 3:**
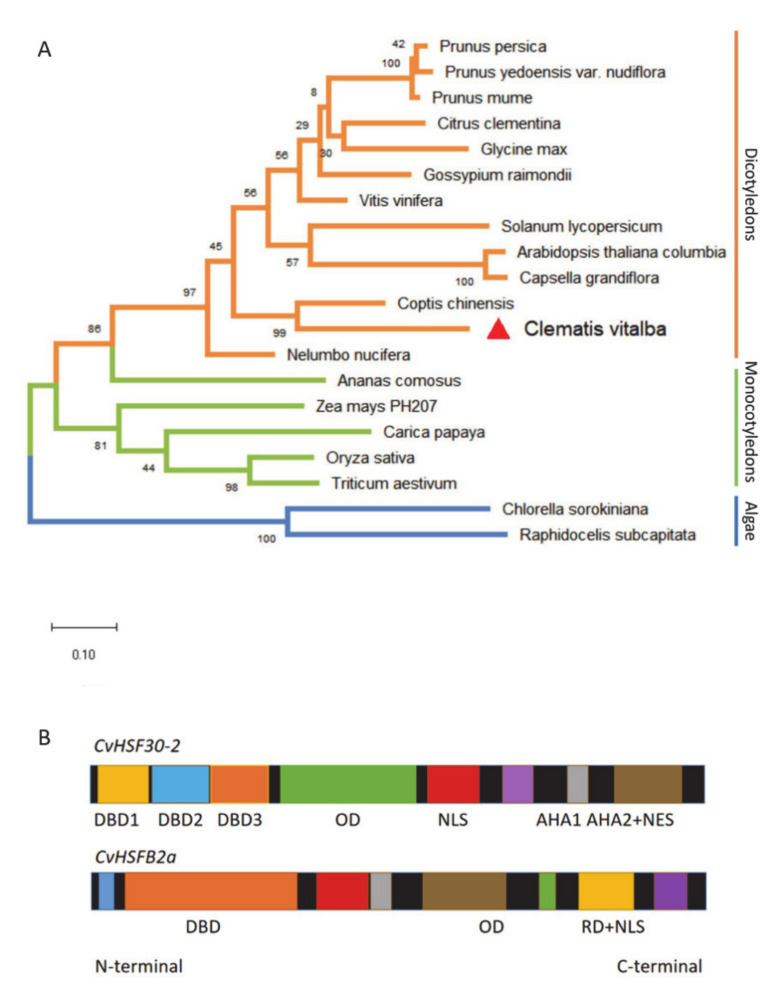
Phylogenetic and motifs analysis of heat shock transcription factors (HSFs). (**A**) Phylogenetic analysis of HSF30-2 in different species. The accession numbers of HSFs are shown top to bottom as follows: XP_008220492.1, Prupe.1G410400.7, PQM38437.1, NP_001290015.1, Gorai.013G220400.5, Ciclev10008618m, Glyma.14G096800.3, Solyc08g062960.2.1, AT2G26150.1, Cagra.7662s0001.1, XP_010262066.1, KAF9613066.1, Cv, Aco005862.1, Zm00008a004344_T01, evm.model.supercontig_44.122, LOC_Os07g08140.1, Traes_2DS_B6872CB84.1, PRW33971.1 and GBF94047.1. (**B**) Motifs of CvHSF30-2 and CvHSFB2a. Motif prediction and visualization were performed in HEATSTER (https://applbio.biologie.uni-frankfurt.de/hsf/heatster/home.php (accessed on 25 July 2020)).

With the combined the RNA-seq and qPCR analysis, *CvHSF30-2* was inferred as a hub gene that may play a critical role in the heat tolerance of *Clematis vitalba*. For further functional characterization of *CvHSF30-2*, we conducted phylogenetic analysis of HSFs from different organisms and found that HSFs can be divided into three groups: those from dicotyledons, monocotyledons and algae, respectively (Figure 2A). CvHSF30-2 was widely dissimilar to other HSF genes available in GenBank and had the highest homology with *Vitis vinifera* (Figure 3A). Motif prediction revealed that CvHSF30-2 had a DNA binding domain (DBD), oligomerization domain (OD), nuclear localization signal (NLS), two activator peptide motifs (AHA motifs) and a nuclear export signal (NES; also functions as a type-specific signature region in the C-terminus of class A HSFs) (Figure 3B), indicating that CvHSF30-2 is a typical type-A heat shock transcription factor.

### 2.4. CvHSF30-2 Enhances the Viability of Tobacco to Survive in Thermal Stress

**Figure 4:**
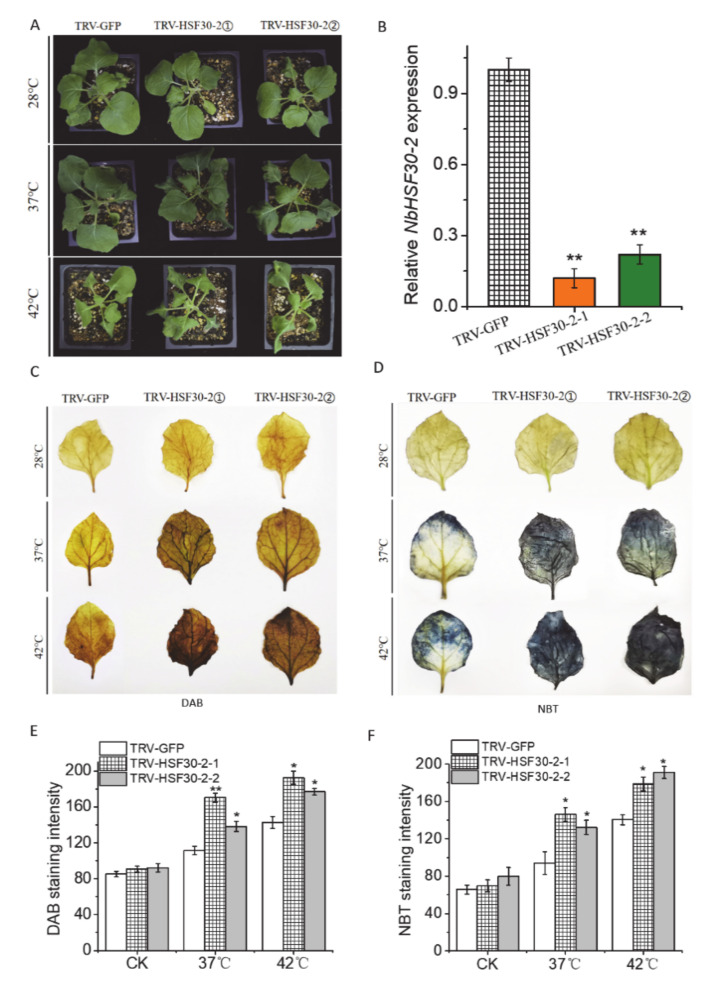
Phenotype of *HSF30-2* silencing in tobacco under heat stress. (**A**) Phenotype of *HSF30-2*-silencing in tobacco by virus-induced gene silencing (VIGS) before and after heat shock. TRV-*HSF30-2* means the silencing of the *CvHSF30-2* homologous gene in tobacco. TRV-HSF30-2① and TRV-HSF30-2② represent two different *HSF30-2*-silencing lines. (**B**) Silencing effect of TRV-*HSF30-2* plants based on qPCR. (**C**,**D**) DAB and NBT staining of *HSF30-2*-silenced tobacco after heat treatment. (**E**,**F**) DAB and NBT staining quantified using the ImageJ2x program. The unit on the x-axis is the average grey value. The staining intensity was quantified using the ImageJ2x program. The image (inverse phase) was changed into an 8-bit grey image, and the average grey values from the upper, middle and lower areas of root tips calculated by ImageJ2x reflect the staining intensity. Error bars represent the standard error of three biological replicates. * *p* < 0.05; ** *p* < 0.01. TRV: tobacco rattle virus; CK: control check (normal temperature).

CvHSF30-2 was chosen to verify whether it can endow plant hosts with high-temperature resistance. We used TRV-based VIGS to silence the homologous genes of *CvHSF30-2* in *N. benthamiana* (Niben101Scf01063g03005). *HSF30-2* expression in newly emerged leaves of *N. benthamiana* significantly decreased after inoculation (Figure 4B). After 37 °C treatment, the TRV-*NbHSF30-2* transgenic line showed a perfect heat sensitive phenotype compared with TRV-GFP (Figure 4A). Heat stress of 42 °C showed a more severe restraint on tobacco. However, TRV-*NbHSF30-2* plants still exhibited a greater wilting phenotype (Figure 4A). Furthermore, more intense DAB and NBT staining was observed in the leaves of TRV-*NbHSF30-2* plants than TRV-GFP after 37 °C and 42 °C treatment (Figure 4C,D); that is, the contents of superoxide dismutase and peroxidase in TRV-*NbHSF30-2* were higher than those in TRV-GFP. The staining intensity was accurately quantified by the ImageJ2x program with inverse phase of the imagers; the higher the grey value, the deeper the DAB/NBT staining. After heat treatment, the grey value was significantly higher in TRV-*NbHSF30-2* plants compared with the control (Figure 4E,F). The result indicated that the silencing of *NbHSF30-2* improved heat sensitivity in *N. benthamiana*.

### 2.5. CvHSF30-2 Confers Tolerance to Heat Stress in Clematis vitalba Plants

**Figure 5:**
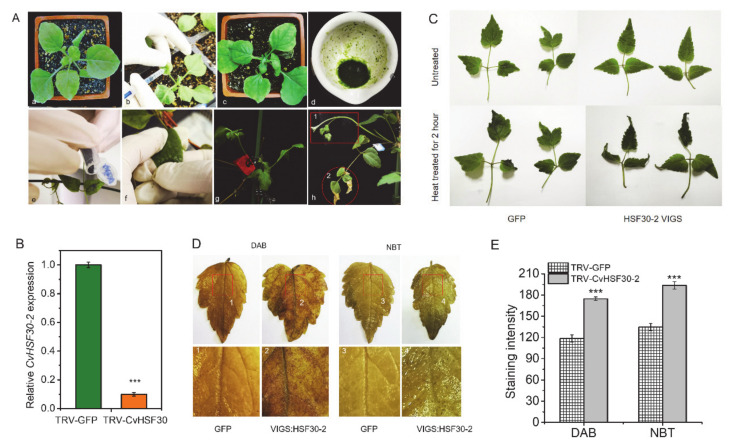
Silencing of *CvHSF30-2* on *Clematis.* (**A**) Operation flow chart of VIGS in Cv. (**a**–**c**) Four-week-old tobacco, *N. benthamiana*, was injected with GV3101 harboring TRV2-*CvHSF30-2*, and the leaves were taken three days later. (**d**–**f**) The leaves injected with TRV2-NBHSF30-2 virus were thoroughly ground. Then, juice was taken and placed on the clematis leaves with quartz sand added. The clematis leaves were inoculated by rubbing by hand. (**g**,**h**) The clematis inoculated by friction was marked and placed in a constant-temperature incubator for cultivation until new branches and leaves had grown. (**B**) Silencing effect of *CvHSF30-2* in control (GFP) and virus-silenced (VIGS: HSF30-2) clematis leaves based on qPCR. (**C**) Heat-shock phenotype of *CvHSF30-2-*silenced Cv by VIGS. (**D**) Staining of Cv leaves after heat shock by DAB and NBT. DAB- and NBT-stained pictures of each group of clematis leaves; the parts of each picture marked with red squares and labelled 1, 2, 3 and 4 correspond to the enlarged pictures below. (**E**) DAB and NBT staining quantified using the ImageJ2x program. Error bars represent the standard error of three biological replicates. *** *p* < 0.001.

In order to further study the function of *CvHSF30-2* in heat response, VIGS technology was used to silence *CvHSF30-2* in Cv. Since the leaf structure of Cv is not suitable for injection inoculation, virus-containing tobacco juice was used to infect *Clematis.* After friction inoculation, Cv plants were cultured for about 20 days (Figure 5A). The newly emerged leaves were treated with 42 °C heat shock for 2 h. The silencing effect of *CvHSF30-2* in Cv leaves was detected by qPCR. *CvHSF30-2* expression decreased in newly emerged leaves of Cv (by about two-thirds) after inoculation, which verified the success of virus infection and silencing application in *Clematis* (Figure 5B). After heat shock, it could be seen that the virus-silenced leaves of Cv showed obvious wilting and curling phenomena, but there was no significant difference in the control group (Figure 5C), indicating that the function of *CvHSF30-2* positively regulated heat tolerance in Cv. Then, DAB and NBT staining showed that the leaves of TRV-*HSF30-2* had obvious staining spots compared with the control group (Figure 5D,E), suggesting its higher ROS accumulation than that of TRV-GFP.

### 2.6. CvHSF30-2 Improves Heat Tolerance of Clematis vitalba by Elevating HSP Expression

**Figure 6:**
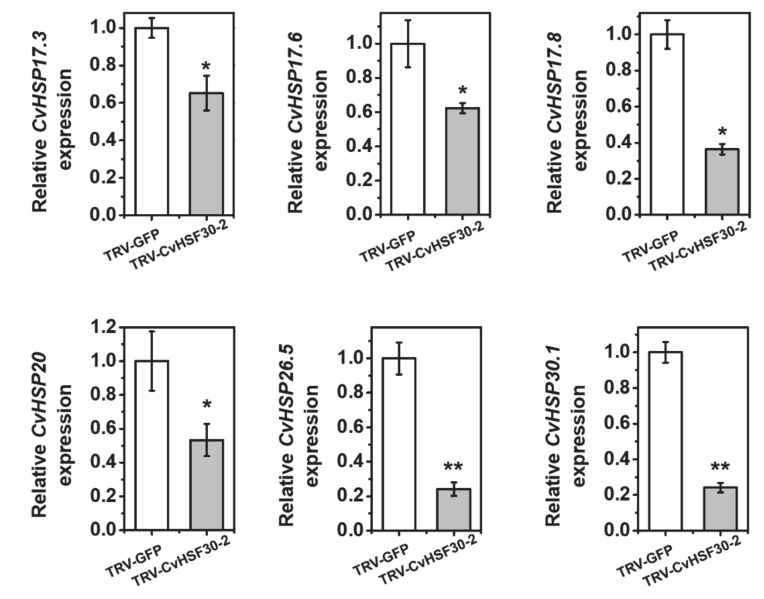
Expressions of *CvHSP17.3*, *CvHSP17.6*, *CvHSP17.8*, *CvHSP20*, *CvHSP26.5* and *CvHSP30.1* in *CvHSF30-2*-silenced plants by VIGS. Error bars represent the standard error of three biological replicates. Asterisks indicate a significant difference between TRV-GFP and TRV-CvHSF30-2 by t-test. * *p* < 0.05; ** *p* < 0.01.

Under transcriptional control of heat shock transcription factors, HSPs respond rapidly to heat stress and stabilize, renature or help to degrade unfolded proteins [31]. Since heat shock transcription factor *CvHSF30-2* was greatly induced after heat shock in *Clematis vitalba*, we speculated that the expressions of HSPs were activated by *CvHSF30-2* and found that HSPs were highly expressing in *Clematis vitalba* when suffering heat shock (Table 1). Furthermore, it has been reported that HSFs function upstream of HSPs [32,33]. Thus we tested whether the expressions of HSPs were affected in TRV-*CvHSF30-2* plants. The result showed that the silencing of *CvHSF30-2* by VIGS led to a significant decrease in *CvHSP17.3*, *CvHSP17.6*, *CvHSP17.8*, *CvHSP20*, *CvHSP26.5* and *CvHSP30.1* expression (Figure 6), which suggested that these HSPs may be positively regulated by *CvHSF30-2* in *Clematis vitalba.*

### 2.7. CvHSFB2a May Be the Negative Regulator for CvHSF30-2

**Figure 7:**
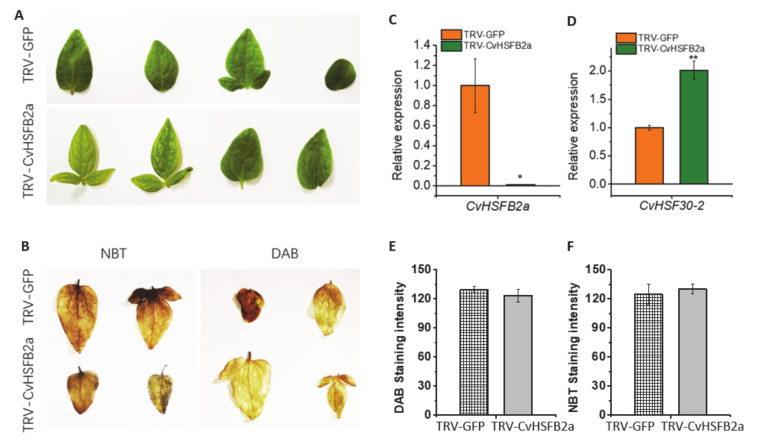
Silencing of *CvHSFB2a* in *Clematis*. (**A**) Phenotype of *CvHSFB2a*-silenced *Clematis* under heat treatment. (**B**) DAB and NBT staining of TRV-GFP and TRV-CvHSFB2a *Clematis* before and after heat treatment. (**C**) Silencing effect of CvHSFB2a. (**D**) The expression of *CvHSF30-2* in TRV-CvHSFB2a plants. (**E**,**F**) DAB and NBT staining as quantified using the ImageJ2x program. Error bars represent the standard error of three biological replicates. * *p* < 0.05; ** *p* < 0.01.

It has been reported that HsfB TFs function as negative regulators of some members of the HsfA family [21]. To compare differences between class A and B HSFs and research the function of class B HSFs, CvHSFB2a was also selected as a representative of class B for further study. Motif prediction revealed that CvHSF30-2 and CvHSFB2a were similar in N-termini while different in C-termini, which indicated a functional difference of the two HSFs. As classical class A and B HSFs, CvHSF30-2 had two activator peptide motifs (AHA motifs) and a nuclear export signal all located in the C-terminus, while CvHSFB2a had a repressor domain (RD) (Figure 3B). In order to investigate the role of CvHSFB2a in regulating CvHSF30-2, we also established a VIGS method for clematis *CvHSFB2a*. Following friction infection of clematis leaves, heat treatment was conducted after the new leaves grew. Both phenotype and staining proved that the heat resistance of the plants did not change significantly (Figure 7A,B,E,F). In the *CvHSFB2a*-silenced *Clematis* plant, the expression of the *CvHSFB2a* gene was obviously reduced (Figure 7C), while the expression of *CvHSF30-2* was significantly increased (Figure 7D), which further demonstrated that CvHSFB2a inhibited the expression of *CvHSF30-2*.

## 3. Discussion

How to resist high temperatures is an important problem for the plants. Compared to traditional model plants such as *Arabidopsis* and rice, research seldom focuses on the heat-tolerant mechanisms of horticultural plants, especially of those without reference genomes. In our study, one heat-tolerant *Clematis* species, *Clematis vitalba*, was identified based on physiological indexes (Figure 1). Transcriptome analysis revealed differentially expressed HTGs, involving heat signal transduction, transcription regulation and protein homeostasis, which have been reported as participating in heat tolerance [31,34]. Among these HTGs, *CvHSF30-2* was highly induced after heat treatment in *Clematis vitalba*, compared with Stolwijk Gold (Figure 2). Further functional verification showed that the overexpression of *CvHSF30-2* conferred resistance to heat stress in *E. coli* and yeast, while the silencing of *HSF30-2* by VIGS led to an enhanced thermosensitivity in tobacco and *Clematis* (Figure 4 and Figure 5). These results confirmed that highly expressing HSFs may be the main reason for thermotolerance in *Clematis vitalba*.

Following heat shock, the response is strictly modulated by heat shock factors and heat shock proteins to inhibit stress injury [35]. Heat shock transcription factors are the vital regulators of the plant heat response. Several HSFs in *Arabidopsis*, such as HSFA2; HSF A1a, A1b, A1d and A1e; HSFA3; HSFA6b or HSFB1; and B2b, can obviously affect plant heat tolerance [21,36]. Heat shock proteins also play an important role in response to thermal stress. Heat treatment greatly increased *HSP90* expression, which directly interacted with transport inhibitor response 1 (TIR1) to prevent its degradation and modulate auxin-mediated plant growth [37,38]. It has been reported that HSFs function upstream of HSPs in heat response [32,33]. Heat signals can activate the transcription activity of the *HSFA1s*, which further regulates the expression of heat-responsive genes such as *HSP70* to enhance plant heat resistance [39]. HSFA2 is required to maintain high levels of specific histone modifications at the 50 regions of *APX2*, *HSP22* and *HSP18.2* [40]. In this study, HSPs were also highly expressing in *Clematis vitalba* when suffering heat shock (Table 1). Additionally, the silencing of *CvHSF30-2* by VIGS led to a significant decrease of *CvHSP17.3*, *CvHSP17.6*, *CvHSP17.8*, *CvHSP20*, *CvHSP26.5* and *CvHSP30.1* expression (Figure 6), which suggested that these HSPs may be positively regulated by *CvHSF30-2* in *Clematis vitalba.* Yeast one-hybrid and electrophoretic mobility shift assay (EMSA) analysis should be performed to gain a further understanding of the regulatory relationship between *CvHSF30-2* and candidate *CvHSPs*.

The model plant *Arabidopsis* contains 21 HSF homologs, which are sorted into three classes (classes A, B and C; [41]). The heat-inducible B-type HSFs, lacking the transactivation domain, may play a different role from HsfA [41]. AtHsfB1 and AtHsfB2b can directly bind to the promoter of HsfA2, as indicated by yeast one-hybrid assay. When transiently coexpressed with *AtHsfA4a*, AtHsfB1 could restrain the transcription of *AtHsfA2* and heat-induced marker genes [21]. Thus, it seemed that HsfB functions as a negative transcription coactivator of some HsfA members. In our study, when *CvHSFB2a* was silenced by VIGS, the expression of *CvHSF30-2* gene was significantly increased (Figure 7C,D), indicating the inhibitor effect of CvHSFB2a in regulating *CvHSF30-2*. Interestingly, no significant heat-sensitive phenotype was observed for the *CvHSFB2a* knockdown line, which was consistent with a previous study of AtHsfB1 [21]. The T-DNA knockout the *HsfB1* line did not show a heat-resistant defective phenotype, and the expressions of some HSPs were not substantially influenced during heat stress, which is probably due to the functional redundancy of AtHsfB2a [42]. Class B HSFs also function as positive regulators of heat response. The tomato heat stress transcription factor HsfB1 cooperated with HsfA1a by recruiting histone acetyl transferase HAC1 and activated HS-inducible genes [13,43]. Additionally, tomato HsfB1 could maintain or restore the expression of housekeeping genes in heat-stressed cells by cooperation with other transcriptional activators [43,44]. In *Arabidopsis*, HsfB1 and HsfB2b significantly promote acquired thermotolerance, probably by repressing HSPs that interfere with the nuclear migration of HSFA1s [21]. Thus, it seems that class B HSFs play a complex and important role in modulating the plant heat response. Future work on the interaction of between CvHSFB2a and class A HSFs might help us better understand the function of HSFB transcription factors in response to heat stimuli.

VIGS is a powerful tool for the study of gene function in plants and has been successfully used in many plants, e.g., *N. benthamiana* [45], wheat [46], tomato [47], *Phalaenopsis* [29] and rose [30]. In this study, we established a method for *Clematis* VIGS by tobacco rattle virus-based vectors. Briefly, TRV was first injected into leaves of *N. benthamiana* for proliferation, and then ground leaf juice was used to infect clematis leaves by friction with quartz sand. The newly growing leaves were used for silencing effect identification and heat tolerance access (Figure 4 and Figure 5). This procedure was similar to the reported tomato VIGS method [47,48]. VIGS integrating intermediate host and friction infection may be a strategy for gene function verification in plants that are difficult to infect by injection. More applications on other plants should be further conducted to confirm its practicability.

**Figure 8:**
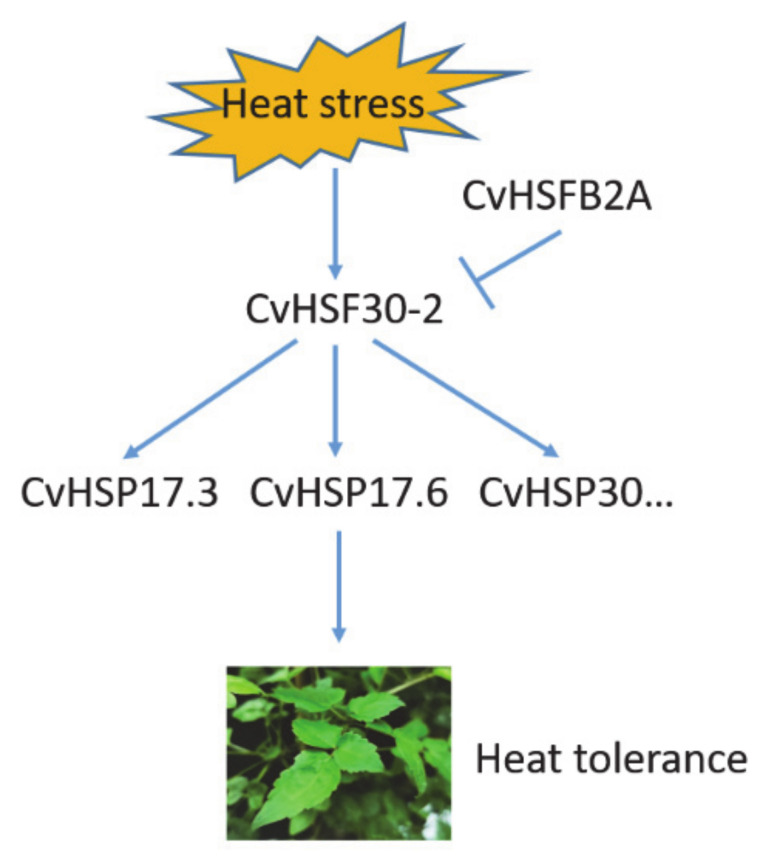
Working model for *Clematis vitalba* thermotolerance.

We developed a hypothetical working model for *Clematis vitalba* thermotolerance based on the results of the current study (Figure 8). When *Clematis* plants are exposed to heat stress, heat shock transcription factor *CvHSF30-2* expression is greatly induced. CvHSF30-2 subsequently elevates the expressions of its candidate downstream HSP targets, e.g., *CvHSP17.3*, *CvHSP17.6* and *CvHSP30*, and then leads to the tolerance of *Clematis* plants in response to heat stress. CvHSFB2a may be the negative regulator of CvHSF30-2 in this process.

## 4. Materials and Methods

### 4.1. Plant Material Growth Conditions and High-Temperature Treatment Conditions

Triennial potted plants of two *Clematis* varieties (*Clematis vitalba* (S-0938 (FR-07)) and Stolwijk Gold) were used in this study. The seeds were derived from Jardin Botanique in France, sown into autoclaved nutrient soil and grown for three years in 12-inch pots in a greenhouse of Shanghai Botanical Garden. For each species, six plants in great and similar growth conditions were taken and placed in two constant-temperature incubators (one at 42 °C, the other at 22 °C) for 2 h, respectively (three plants per constant-temperature incubator). Treated potted plants were used for the subsequent analysis and RNA-seq of the physiological indexes.

### 4.2. Measurement of Physiological Indexes before and after High-Temperature Treatment

Relative conductivity: 0.2 g of leaves was placed in a 20 mL tube, vacuumized and left to stand for 30 min at normal temperature (the tube was shaken gently every 5 min during the period). The conductivity was measured with a DDS-11A-type conductivity meter. After a boiling water bath for 10 min and cooling, the conductivity value was again measured and the relative conductivity was calculated. The relative conductivity per gram of fresh weight was used to represent relative conductivity. Measuring relative water content: 0.2 g of chopped leaves was weighed and placed in a weighing bottle, then put in an oven for 30 min at 105 °C for dehumidification. The oven was then set to 80 °C for drying to constant weight, the sample was taken out and cooled to normal temperature, and the dry weight was weighed and the relative water content calculated.

### 4.3. Nitro Blue Tetrazole and Diaminobenzidine Staining before and after High-Temperature Treatment

In DAB staining, leaves of two *Clematis* varieties subjected to normal or high-temperature treatments were soaked in DAB staining solution at 25 °C for 24 h in the dark, and then soaked in 95% ethanol to remove chlorophyll. In NBT staining, the leaves of two *Clematis* varieties subjected to normal or high-temperature treatments were immersed in the NBT staining solution at 25 °C for 12 h in the dark, and then immersed in 95% ethanol to remove chlorophyll.

### 4.4. RNA Extraction and Quantitative PCR Assay

The young leaves (three- to four-weeks-old, heat-treated or untreated controls) of *Clematis* plants were harvested for the detection of heat-related gene expressions. The newly growing leaves of control and VIGS tobacco (two-weeks-old) or *Clematis* plants (three- to four-weeks-old) were harvested for silencing effect identification. Total RNA was extracted using a SteadyPure Plant RNA Extraction Kit (Code: AG21019; Accurate Biotechnology Co., Ltd., Hunan, China). cDNA synthesis was performed with the RT reagent kit (Takara) according to the manufacturer’s protocol. Real-time PCRs were done on a Chromo 4™ continuous fluorescence detector with the SYBR RT-PCR Kit (Takara) in a 20 μL reaction volume, which contained 10 μL of SYBR Green I PCR mix, 0.5 μM each of forward and reverse primer, 1 μL of cDNA template and appropriate amounts of sterile ddH_2_O. Amplification conditions were: 2 min at 95 °C; 40 cycles of 15 s at 95 °C, 30 s at 58 °C and 30 s at 72 °C. Fold changes of RNA transcripts were calculated by the 2^−ΔΔCt^ method [49]. We used *CvUBC2D* (TRINITY_DN89495_c0_g1) in *Clematis* plants and *NbEF1α* in tobacco as the internal controls. The entire experiments were repeated three times. Primers used in the qPCR are listed in Supplementary Table S7.

### 4.5. RNA-Seq Data Processing, Reassembly and Annotation

RNA purification, reverse transcription, library construction and sequencing were performed at Shanghai Majorbio Bio-pharm Biotechnology Co., Ltd. (Shanghai, China) according to the manufacturer’s instructions (Illumina, San Diego, CA, USA). The RNA-seq transcriptome libraries were prepared using an Illumina TruSeqTM RNA Sample Preparation Kit (San Diego, CA, USA). RNAseq libraries were sequenced in single lane on an Illumina Hiseq xten/NovaSeq 6000 sequencer (Illumina, San Diego, CA, USA) for 2 × 150 bp paired-end reads. The RNA-seq raw read data was processed using the software fastx_toolkit_0.0.14 (http://hannonlab.cshl.edu/fastx_toolkit/ (accessed on 5 May 2020)), SeqPrep (https://github.com/jstjohn/SeqPrep (accessed on 5 May 2020)) and Sickle (https://github.com/najoshi/sickle (accessed on 5 May 2020)) to evaluate and discard sequences of low quality and those affected by adaptor contamination, with quality control shown in Supplementary Tables S3 and S4. After obtaining high-quality RNA-seq sequencing data, Trinity (Version v2.8.5, https://github.com/trinityrnaseq/trinityrnaseq (accessed on 5 May 2020)) was used to assemble all clean data from scratch due to the lack of a reference genome for *Clematis*. Then, the assembly results were optimized with TransRate (http://hibberdlab.com/transrate/ (accessed on 5 May 2020)) and CD-HIT (http://weizhongli-lab.org/cd-hit/ (accessed on 5 May 2020)). BUSCO (Benchmarking Universal Single-Copy Orthologs, http://busco.ezlab.org (accessed on 5 May 2020)) was used for a re-evaluation of the optimized sequences. The assembly quality is shown in Supplementary Table S5. The assembled high quality transcriptome sequences were annotated by six major databases (NR, Swiss-Prot, Pfam, COG, GO and KEGG databases). The versions and sources of the software and databases we used are shown in Supplementary Table S6.

### 4.6. Functional Verification of VIGS with Clematis or Tobacco as Host

For the tobacco VIGS assay [24], part of the homologous nucleotide sequence of *CvHSF30-2* in *N. benthamiana* was amplified using the primers in Supplementary Table S7 and determined by Sangon Biotech Co., Ltd. (Shanghai, Chana). Then, the fragments were separately linked to the pTRV2 virus vector. The constructed pTRV2 vectors mentioned above were introduced into the *A. tumefaciens* strain GV3101. *Agrobacterium* harboring TRV1- or TRV2-derived vectors was mixed in a 1:1 ratio and infiltrated into the leaves of two-week-old *N. benthamiana* plants. The newly growing leaves were used for heat tolerance access and silencing effect identification two weeks after injection.

For the *Clematis* VIGS assay, 246 bp in the 5′-region of *CvHSF30-2* mRNA were amplified using the primers in Supplementary Table S7 and determined by Sangon Biotech Co., Ltd. (Shanghai, China). Then, the fragments were separately linked to the pTRV2 virus vector. *A. tumefaciens* strain GV3101 harboring TRV1- or TRV2-derived vectors was mixed in a 1:1 ratio and infiltrated into the leaves of four-week-old *N. benthamiana* plants as the intermediate hosts. Three days after inoculation, the inoculated tobacco leaves were ground into juice and inoculated into *Clematis* by friction with quartz sand. After inoculation, the plants were cultivated at 20–25 °C for three to four weeks. The silencing effect of the target gene was detected on every branch using newly growing apical leaves. The appropriate newly growing leaves on the same branch were used for heat tolerance assessment.

## Figures and Tables

**Table 1:** Differently expressing heat shock proteins (HSPs) before and after heat treatment (*p* < 0.05).

Gene_id	Gene Name	Fold Change	P-Adjust	NR Description
TRINITY_DN2523_c0_g1	CvHSP17.3	18.24959617	5.84 × 10^−72^	Predicted: 17.3 kDa class I heat shock protein-like (*Ipomoea nil*)
TRINITY_DN55579_c0_g1	CvHSP17.6	15.30769231	0.149868384	Predicted: 17.6 kDa class I heat shock protein 1-like (*Solanum tuberosum*)
TRINITY_DN28316_c0_g1	CvHSP30-1	8.297101449	0.010914936	30 kDa heat shock protein-like (*Quercus suber*)
TRINITY_DN19999_c0_g1	CvHSP30-2	7.306338028	0.022396861	30 kDa heat shock protein-like (*Quercus suber*)
TRINITY_DN1775_c1_g2	CvHSP17.8	5.753496246	7.18 × 10^−36^	Predicted: 17.8 kDa class I heat shock protein-like (*Daucus carota* subsp. *sativus*)
TRINITY_DN316_c0_g2	CvHSP70-1	4.37847865	2.61 × 10^−16^	Heat shock protein 70 (*Chimonanthus praecox*)
TRINITY_DN60388_c0_g1	CvHSP83	4.170731707	0.014590875	Predicted: heat shock protein 83-like (*Nelumbo nucifera*)
TRINITY_DN3156_c0_g1	CvHSP70-2	4.145122415	1.61 × 10^−13^	HSP70 domain-containing protein (*Cephalotus follicularis*)
TRINITY_DN1775_c0_g2	CvHSP20	3.12634968	0.002560726	Alpha crystallin/Hsp20 domain (*Macleaya cordata*)
TRINITY_DN11240_c0_g1	CvHSP26.5	3.125766871	0.051866641	26.5 kDa heat shock protein, mitochondrial (*Sesamum indicum*)

## Data Availability

RNA-seq reads are available at https://submit.ncbi.nlm.nih.gov/subs/sra/ (accessed on 17 January 2021) with accession number PRJNA692678.

## Supplementary Materials

The Supplementary Materials for this article can be found at https://www.mdpi.com/1422-0067/22/6/2900/s1. Figure S1: PCA and Venn analysis of Cv and SG, Table S1: A total of 58 differentially expressing transcription factors were found in Cv before and after heat treatment, Table S2: Description of differently expressing heat-related genes shown in the heat map, Table S3: Quality control of Cv transcriptome analysis, Table S4: Quality control of SG transcriptome analysis, Table S5: The parameters of the transcriptome assembly with Trinity, Table S6: The versions and sources of the software/databases used in transcriptome analysis, Table S7: Primers used in this study.
