# Supplementary material for: One Heat Shock Transcription Factor Confers High Thermal Tolerance in Clematis Plants
Source: Int J Mol Sci. 2021 Mar 12;22(6):2900. doi: 10.3390/ijms22062900 (PMC7998627; doi:10.3390/ijms22062900)
Supplement: Supplementary file 1 [file ijms-22-02900-s001.zip › Supplementary File-8/Supplementary File-8.docx]

**Supplementary File**


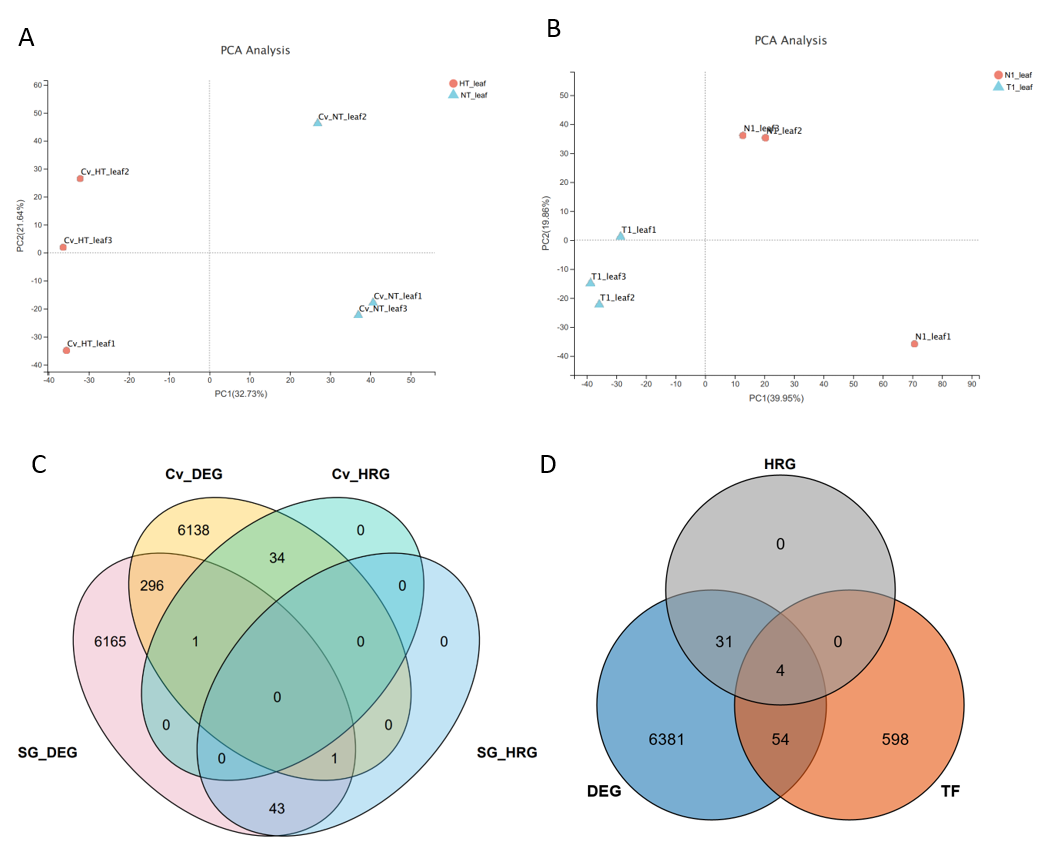


**FIGURE. S1 PCA and Venn analysis of CV and SG. PCA analysis of CV (A) and SG (B) before and after heat shock. (C) Venn analysis of DEGs (differential expressing genes) and HRG (heat-related genes) in Cv and SG. (D) Venn analysis of DEG, TF, and HRG in Cv after heat shock.**

**Table. S1 58 differentially expressing transcription factors in CV before and after heat treatment.**

| **Gene_id** | FC(HT_leaf/NT_leaf) | Pvalue | **Regulate** | **NR description** |
| --- | --- | --- | --- | --- |
| TRINITY_DN22089_c0_g1 | 0.245858477 | 1.12E-31 | down | OVA00458.1(cAMP response element binding (CREB) protein [Macleaya cordata]) |
| TRINITY_DN15356_c0_g1 | 0.267620997 | 1.02E-24 | down | PIA60627.1(hypothetical protein AQUCO_00300259v1 [Aquilegia coerulea]) |
| TRINITY_DN42421_c0_g2 | 0.218156326 | 6.84E-18 | down | PIA64338.1(hypothetical protein AQUCO_00100071v1 [Aquilegia coerulea]) |
| TRINITY_DN45782_c0_g1 | 0.275923931 | 2.11E-15 | down | XP_028948914.1(protein FAR1-RELATED SEQUENCE 5-like [Malus domestica]) |
| TRINITY_DN4701_c0_g1 | 0.134897721 | 3.39E-15 | down | XP_021596550.1(putative GATA transcription factor 22 [Manihot esculenta] ) |
| TRINITY_DN67628_c0_g1 | 893.756619 | 1.75E-13 | up | XP_012093009.1(NAC domain-containing protein 100 [Jatropha curcas] ) |
| TRINITY_DN68252_c0_g1 | 0.328231153 | 9.55E-13 | down | OVA10986.1(Transcription factor [Macleaya cordata]) |
| TRINITY_DN66329_c0_g1 | 0.106989158 | 1.62E-11 | down | PIA61158.1(hypothetical protein AQUCO_00300583v1 [Aquilegia coerulea]) |
| TRINITY_DN29112_c0_g1 | 0.272853834 | 1.92E-11 | down | PIA65555.1(hypothetical protein AQUCO_00100803v1 [Aquilegia coerulea]) |
| TRINITY_DN45023_c0_g2 | 0.312528018 | 6.76E-11 | down | PIA39637.1(hypothetical protein AQUCO_02600236v1 [Aquilegia coerulea]) |
| TRINITY_DN67693_c0_g1 | 0.214851346 | 5.46E-09 | down | PIA62640.1(hypothetical protein AQUCO_00200568v1 [Aquilegia coerulea]) |
| TRINITY_DN17680_c0_g2 | 4.084731705 | 4.16E-08 | up | PIA50076.1(hypothetical protein AQUCO_01300661v1 [Aquilegia coerulea]) |
| TRINITY_DN4826_c0_g1 | 0.278329915 | 2.97E-06 | down | PIA32710.1(hypothetical protein AQUCO_04400122v1 [Aquilegia coerulea]) |
| TRINITY_DN56957_c0_g1 | 4.111095718 | 1.02E-05 | up | PIA36451.1(hypothetical protein AQUCO_03400376v1 [Aquilegia coerulea]) |
| TRINITY_DN9250_c0_g1 | 32.15407685 | 0.00010918 | up | EOY06018.1(Integrase-type DNA-binding superfamily protein [Theobroma cacao]) |
| TRINITY_DN21191_c0_g2 | 134.633566 | 0.000229327 | up | PIA29933.1(hypothetical protein AQUCO_05800185v1 [Aquilegia coerulea]) |
| TRINITY_DN9745_c0_g1 | 3.56378608 | 0.000300915 | up | PIA37535.1(hypothetical protein AQUCO_03000245v1 [Aquilegia coerulea]) |
| TRINITY_DN10817_c1_g1 | 3.093525373 | 0.000376965 | up | XP_010244980.1(PREDICTED: dof zinc finger protein DOF3.2-like [Nelumbo nucifera]) |
| TRINITY_DN9948_c0_g5 | 0.275585984 | 0.00040539 | down | PIA51834.1(hypothetical protein AQUCO_01000012v1 [Aquilegia coerulea]) |
| TRINITY_DN1120_c0_g1 | 0.273350089 | 0.000808696 | down | XP_022135702.1(transcription factor WER-like [Momordica charantia]) |
| TRINITY_DN7531_c0_g1 | 4.545526235 | 0.000986695 | up | PIA50119.1(hypothetical protein AQUCO_01300693v1 [Aquilegia coerulea]) |
| TRINITY_DN36188_c0_g1 | 42.03982267 | 0.001130014 | up | PIA35114.1(hypothetical protein AQUCO_03600050v1 [Aquilegia coerulea]) |
| TRINITY_DN57689_c0_g1 | 8.139476556 | 0.001262127 | up | PIA64533.1(hypothetical protein AQUCO_00100189v1 [Aquilegia coerulea]) |
| TRINITY_DN16888_c0_g3 | 3.506935268 | 0.001371264 | up | PIA62741.1(hypothetical protein AQUCO_00200639v1 [Aquilegia coerulea]) |
| TRINITY_DN8826_c0_g1 | 9.940079941 | 0.001418321 | up | PIA44227.1(hypothetical protein AQUCO_01700082v1 [Aquilegia coerulea]) |
| TRINITY_DN3971_c0_g1 | 9.70503095 | 0.002004653 | up | XP_010240843.1(PREDICTED: agamous-like MADS-box protein AGL80 [Nelumbo nucifera]) |
| TRINITY_DN13741_c0_g2 | 9.321012529 | 0.002312686 | up | PIA48547.1(hypothetical protein AQUCO_01400855v1 [Aquilegia coerulea]) |
| TRINITY_DN34303_c0_g1 | 14.72406959 | 0.003298885 | up | PIA27591.1(hypothetical protein AQUCO_07600040v1 [Aquilegia coerulea] ) |
| TRINITY_DN5075_c0_g1 | 0.319659859 | 0.003490356 | down | ACQ82820.1(R2R3-myb transcription factor [Aquilegia formosa]) |
| TRINITY_DN27856_c0_g1 | 40.9908677 | 0.003525652 | up | PIA33443.1(hypothetical protein AQUCO_04100103v1 [Aquilegia coerulea]) |
| TRINITY_DN5812_c0_g1 | 6.307158898 | 0.003527728 | up | APD70959.1(SPOROCYTELESS-like EAR-containing protein 1 [Aquilegia coerulea] ) |
| TRINITY_DN66129_c0_g1 | 6.747416654 | 0.006673422 | up | XP_008453188.1(PREDICTED: transcription factor PRE3-like [Cucumis melo]) |
| TRINITY_DN13074_c0_g1 | 0.304984185 | 0.007295303 | down | PIA40986.1(hypothetical protein AQUCO_02300039v1 [Aquilegia coerulea]) |
| TRINITY_DN18034_c0_g2 | 4.665719508 | 0.007296994 | up | XP_010917172.1(transcription repressor MYB6-like [Elaeis guineensis]) |
| TRINITY_DN32931_c0_g1 | 4.52555985 | 0.0076358 | up | PIA50118.1(hypothetical protein AQUCO_01300692v1 [Aquilegia coerulea]) |
| TRINITY_DN67683_c0_g1 | 8.538519182 | 0.008378651 | up | PIA48510.1(hypothetical protein AQUCO_01400829v1 [Aquilegia coerulea]) |
| TRINITY_DN63034_c0_g1 | 4.405263954 | 0.008870314 | up | PIA27437.1(hypothetical protein AQUCO_07800053v1 [Aquilegia coerulea]) |
| TRINITY_DN15546_c0_g1 | 34.13090161 | 0.0090252 | up | XP_004305612.1(PREDICTED: transcription repressor MYB6-like [Fragaria vesca subsp. vesca]) |
| TRINITY_DN59554_c0_g1 | 7.752539187 | 0.009638963 | up | PIA49226.1(hypothetical protein AQUCO_01300221v1 [Aquilegia coerulea]) |
| TRINITY_DN10764_c0_g1 | 3.415092114 | 0.010864099 | up | PIA26059.1(hypothetical protein AQUCO_10000022v1 [Aquilegia coerulea] ) |
| TRINITY_DN9250_c0_g2 | 12.39443388 | 0.012968194 | up | PIA36452.1(hypothetical protein AQUCO_03400377v1 [Aquilegia coerulea]) |
| TRINITY_DN53229_c0_g1 | 3.690588794 | 0.013784954 | up | PIA37535.1(hypothetical protein AQUCO_03000245v1 [Aquilegia coerulea]) |
| TRINITY_DN66259_c0_g1 | 3.907769537 | 0.014104297 | up | PIA57038.1(hypothetical protein AQUCO_00600040v1 [Aquilegia coerulea]) |
| TRINITY_DN65561_c0_g1 | 21.40777669 | 0.017583162 | up | XP_007019013.1(PREDICTED: ethylene-responsive transcription factor ERF014 [Theobroma cacao] ) |
| TRINITY_DN37555_c1_g1 | 6.007643666 | 0.017598133 | up | XP_008219733.2(PREDICTED: protein FAR1-RELATED SEQUENCE 5-like [Prunus mume]) |
| TRINITY_DN63949_c0_g1 | 0.195847947 | 0.020066461 | down | RWW56791.1(hypothetical protein BHE74_00036474 [Ensete ventricosum]) |
| TRINITY_DN40417_c0_g2 | 4.563162032 | 0.023677083 | up | PIA51732.1(hypothetical protein AQUCO_01100538v1 [Aquilegia coerulea]) |
| TRINITY_DN56961_c0_g1 | 3.05213166 | 0.026604059 | up | PIA34887.1(hypothetical protein AQUCO_03700272v1 [Aquilegia coerulea]) |
| TRINITY_DN39555_c0_g4 | 9.793989734 | 0.032565625 | up | PIA60378.1(hypothetical protein AQUCO_00300104v1 [Aquilegia coerulea]) |
| TRINITY_DN44750_c2_g2 | 8.511015096 | 0.034810598 | up | PIA45312.1(hypothetical protein AQUCO_01700683v1 [Aquilegia coerulea]) |
| TRINITY_DN38896_c0_g1 | 0.070454463 | 0.036018784 | down | PIA58669.1(hypothetical protein AQUCO_00500544v1 [Aquilegia coerulea]) |
| TRINITY_DN22819_c0_g1 | 3.133128214 | 0.036889914 | up | PIA28683.1(hypothetical protein AQUCO_06700006v1 [Aquilegia coerulea]) |
| TRINITY_DN68509_c0_g1 | 3.392278698 | 9.96E-14 | up | XP_010278594.1(PREDICTED: heat stress transcription factor B-2b-like [Nelumbo nucifera]) |
| TRINITY_DN17619_c1_g4 | 3.283405947 | 1.36E-07 | up | PIA46283.1(hypothetical protein AQUCO_01500059v1 [Aquilegia coerulea] ) |
| TRINITY_DN3079_c0_g3 | 12.39590134 | 1.38E-40 | up | XP_010648342.1(PREDICTED: heat shock factor protein HSF30 isoform X1 [Vitis vinifera]) |
| TRINITY_DN3079_c0_g2 | 5.717236755 | 7.83E-23 | up | PIA64295.1(hypothetical protein AQUCO_00100047v1 [Aquilegia coerulea] ) |
| TRINITY_DN6499_c1_g1 | 4.893409564 | 2.36E-07 | up | OVA00587.1(Heat shock factor (HSF)-type [Macleaya cordata]) |
| TRINITY_DN26678_c0_g1 | 9.339897537 | 4.41E-06 | up | PIA64295.1(hypothetical protein AQUCO_00100047v1 [Aquilegia coerulea] ) |

**Table. S2 Description of differently expressing heat-related genes shown in the heat map.**

| Abbreviation | Description |
| --- | --- |
| HSP17.6II | HSP17.6II |
| HSP20-like-1 | HSP20-like chaperones superfamily protein |
| HSP17.6 | ATHSP17.6A HSP17.6 HSP17.6A |
| TMS1 | J domain protein localized in ER lumen, similarity to HSP40 proteins |
| HSP70 | ATHSP70 HSP70 |
| HSP70T-2 | HSP70T-2 |
| RDDP-Like-1 | RNA-directed DNA polymerase (reverse transcriptase)-related family protein |
| DNAJ | dnaJ protein homolog isoform X1 |
| BIP1 | The luminal binding protein BiP, an ER-localized member of the HSP70 family |
| RDDP-Like-2 | RNA-directed DNA polymerase (reverse transcriptase)-related family protein |
| ATJ2 | ATJ2 DNAJ homolog 2 |
| TBL29 | DUF231 domain proteins |
| ESK1 | DUF231 domain proteins |
| Hsp83 | AtHsp90.4 Hsp81.4 |
| HSFB2A | HSFB2A |
| RDDP-Like-3 | RNA-directed DNA polymerase (reverse transcriptase)-related family protein |
| HSP20-like-2 | HSP20-like chaperones superfamily protein |
| RHP | RNA-directed DNA polymerase (reverse transcriptase)-related family protein |
| RDDP-Like-4 | RNA-directed DNA polymerase (reverse transcriptase)-related family protein |
| RHP-Like-1 | Ribonuclease H-like superfamily protein |
| CYSB | Encodes a protein with cysteine proteinase inhibitor activity |
| Hsp40 | Molecular chaperone Hsp40/DnaJ family protein |
| HSC70 | HEAT SHOCK COGNATE PROTEIN 70-1 |
| J2 | DNAJ HOMOLOGUE 2 |
| RDDP-Like-5 | RNA-directed DNA polymerase (reverse transcriptase)-related family protein |
| RHP-Like-2 | Ribonuclease H-like superfamily protein |
| Unknown protein | Unknown protein |
| RDDP-Like-6 | RNA-directed DNA polymerase (reverse transcriptase)-related family protein |
| Cv14-3-3 | GF14 UPSILON, GRF5, a 14-3-3 gene family member |
| RHP-Like--3 | Ribonuclease H-like superfamily protein |
| RDDP-Like-7 | RNA-directed DNA polymerase (reverse transcriptase)-related family protein |
| HSFA2-Like | ATHSFA2 HSFA2 |
| HSF30-2 | AT-HSFA7A HSFA7A |
| HSFB2A-Like | AT-HSFB2A HSFB2A |
| HSFA2 | ATHSFA2 HSFA2 |

**Table. S3 Quality control of CV transcriptome analysis.**

| Sample | Clean reads | Clean bases | Error rate(%) | Q20(%) | Q30(%) | GC content(%) |
| --- | --- | --- | --- | --- | --- | --- |
| Cv_HT_leaf1 | 52170822 | 7790566154 | 0.0116 | 98.42 | 95.63 | 45.4 |
| Cv_HT_leaf2 | 61184264 | 9139904850 | 0.0115 | 98.52 | 95.84 | 45.42 |
| Cv_HT_leaf3 | 53833638 | 8035007446 | 0.0114 | 98.58 | 95.97 | 45.49 |
| Cv_NT_leaf1 | 56857032 | 8488134771 | 0.0116 | 98.48 | 95.74 | 45.46 |
| Cv_NT_leaf2 | 53709870 | 8017766736 | 0.0114 | 98.54 | 95.91 | 45.56 |
| Cv_NT_leaf3 | 56312236 | 8408827513 | 0.0115 | 98.52 | 95.85 | 45.38 |

**Table. S4 Quality control of SG transcriptome analysis.**

| Sample | Clean reads | Clean bases | Error rate(%) | Q20(%) | Q30(%) | GC content(%) |
| --- | --- | --- | --- | --- | --- | --- |
| N1_leaf1 | 50030186 | 7501570878 | 0.0199 | 98.29 | 94.95 | 47.04 |
| N1_leaf2 | 44570666 | 6692966618 | 0.0233 | 98.78 | 95.88 | 45.86 |
| N1_leaf3 | 46475422 | 6979035390 | 0.0233 | 98.77 | 95.83 | 45.81 |
| T1_leaf1 | 45021368 | 6764910150 | 0.0233 | 98.75 | 95.78 | 45.42 |
| T1_leaf2 | 43079186 | 6474341514 | 0.0235 | 98.7 | 95.6 | 45.38 |
| T1_leaf3 | 46565298 | 6999809694 | 0.0235 | 98.7 | 95.61 | 45.8 |

**Table. S5 The parameters of the transcriptome assembly with Trinity.**

| **Type** | **Unigene** | **Transcript** |
| --- | --- | --- |
| Total number | 83790 | 122405 |
| Total base | 79199631 | 132723096 |
| Largest length (bp) | 16938 | 16938 |
| Smallest length (bp) | 201 | 201 |
| Average length (bp) | 945.22 | 1084.29 |
| N50 length (bp) | 1564 | 1717 |
| E90N50 length (bp) | 2480 | 2208 |
| Fragment mapped percent(%) | 72.239 | 80.583 |
| GC percent (%) | 41.26 | 41.27 |
| TransRate score | 0.27458 | 0.34037 |
| BUSCO score | C:79.7%[S:77.3%;D:2.4%] | C:79.7%[S:77.3%;D:2.4%] |

**Table. S6 The version and source of soft/database used in transcriptome analysis.**

| **Soft/Database** | **Version** | **Source** |
| --- | --- | --- |
| fastx_toolkit | Version 0.0.14 | http://hannonlab.cshl.edu/fastx_toolkit/ |
| Sickle | -- | https://github.com/najoshi/sickle |
| SeqPrep | -- | https://github.com/jstjohn/SeqPrep |
| fastp | Version 0.19.5 | https://github.com/OpenGene/fastp |
| TGICL | Version 2.1 | https://sourceforge.net/projects/tgicl/files/latest/download |
| Trinity | Version v2.8.5 | https://github.com/trinityrnaseq/trinityrnaseq |
| SPAdes | Version 3.13.1 | https://github.com/ablab/spades |
| BUSCO | Version 3.0.2 | https://busco.ezlab.org/ |
| cd-hit | Version v4.5.7 | https://github.com/weizhongli/cdhit |
| hisat2 | Version 2.1.0 | http://ccb.jhu.edu/software/hisat2/index.shtml |
| samtools | Version 1.9 | https://github.com/samtools/samtools.git |
| transrate | Version v1.0.3 | http://hibberdlab.com/transrate/index.html |
| RSEM | Version 1.3.1 | http://deweylab.biostat.wisc.edu/rsem/ |
| kallisto | Version 0.46.0 | https://pachterlab.github.io/kallisto/download |
| Salmon | Version 0.14.1 | https://github.com/COMBINE-lab/salmon |
| bowtie2 | Version 2.3.5.1 | https://sourceforge.net/projects/bowtie-bio/files/bowtie2/2.3.5.1/ |
| DESeq2 | Version 1.24.0 | http://bioconductor.org/packages/stats/bioc/DESeq2/ |
| edgeR | Version 3.24.3 | http://bioconductor.org/packages/stats/bioc/edgeR/ |
| DEGSeq | Version 1.38.0 | http://bioconductor.org/packages/stats/bioc/DEGSeq/ |
| misa | version 2.3.6 | http://pgrc.ipk-gatersleben.de/misa/misa.html |
| TransDecoder | Version 5.5.0 | http://transdecoder.github.io/ |
| HMMER | Version 3.2.1 | http://www.hmmer.org/download.html |
| bwa | version 0.7.9a | https://sourceforge.net/projects/bio-bwa/files/ |
| bcftools | version 1.9 | https://github.com/samtools/samtools.git |
| sentieon | -- | -- |
| GATK | Version 3.8 | https://software.broadinstitute.org/gatk/download/ |
| BLAST+ | Version 2.9.0 | ftp://ftp.ncbi.nlm.nih.gov/blast/executables/blast+/2.9.0/ |
| Diamond | Version 0.9.24 | https://github.com/bbuchfink/diamond |
| WGCNA | Version 1.63 | https://horvath.genetics.ucla.edu/html/CoexpressionNetwork/Rpackages/WGCNA/ |
| STEM | Version 1.3.11 | http://www.cs.cmu.edu/~jernst/stem/ |
| maSigPro | Version 1.56.0 | http://www.bioconductor.org/packages/release/bioc/html/maSigPro.html |
| GSEA | Version 3.0 | http://software.broadinstitute.org/gsea/index.jsp |
| KEGG database | Version 2017.08 | http://www.genome.jp/kegg/ |
| eggNOG database | Version 5.0 | http://eggnogdb.embl.de/#/app/home |
| Pfam database | Version v32.0 | http://pfam.xfam.org/ |
| Swiss-prot database | Version 2019.7.1 | ftp://ftp.uniprot.org/pub/databases/uniprot/current_release/knowledgebase/complete/uniprot_sprot.fasta.gz |
| GO database | -- | http://www.geneontology.org/ |
| NR database | Version 2019.6.26 | ftp://ftp.ncbi.nlm.nih.gov/blast/db/ |
| Rfam database | Version Rfam v14.1 | http://rfam.janelia.org/ |
| goatools | Version 0.6.5 | https://files.pythonhosted.org/packages/bb/7b/0c76e3511a79879606672e0741095a891dfb98cd63b1530ed8c51d406cda/goatools-0.8.9.tar.gz |
| MSigDB database | Version 6.2 | http://software.broadinstitute.org/gsea/downloads.jsp |
| STRING database | Version 11.0 | https://string-db.org/ |
| AnimalTFDB database | Version 3.0 | http://bioinfo.life.hust.edu.cn/AnimalTFDB/ |
| PlantTFDB database | Version 4.0 | http://planttfdb.cbi.pku.edu.cn/ |

**Table S7 Primers used in this study.**

| Primer | Sequence |
| --- | --- |
| VIGS for *N. benthamiana* |  |
| NbHSF30-2-TRV2-1-F | TGAGTAAGGTTACCGAATTCCCTCCTTTTCTGAGCAAGA |
| NbHSF30-2-TRV2-1-R | GACATGCCCGGGCCTCGAGTGTCTGACGAAGCTGGAGAA |
| NbHSF30-2-TRV2-2-F | TGAGTAAGGTTACCGAATTCGGTTTCAGGAAGGTTGATCC |
| NbHSF30-2-TRV2-2-R | GACATGCCCGGGCCTCGAGCTGCTGCTGTTTCAACTCAA |
| VIGS for *Clematis* |  |
| CvHSF30-TRV2-F： | TGAGTAAGGTTACCGAATTCTCAAGTGACAACTGGGCAAG |
| CvHSF30-TRV2-R： | GACATGCCCGGGCCTCGAGGGGCAAGTCCCAAACAATAA |
| CvHSFB-TRV2-F | TGAGTAAGGTTACCGAATTCATGACTCCTCCTCCGCCGGTGGAA |
| CvHSFB-TRV2-R | GACATGCCCGGGCCTCGAGCGAATTCCCATCTATCTGGTACAA |
| qPCR |  |
| qCv14.3.3-F | AGGTGTTTTGGTGATTATCATTAT |
| qCv14.3.3-R | AATGCGTAGGAGCAAGTTCCACTG |
| qCvRHP-F | TGCGGATGAGTGTATCCTATGCTG |
| qCvRHP-R | CCCATAAGCTTTCCGCCAGAGTTC |
| qCvdnaJ-F | AGAAGCTTTCCCTCTCCCGAAACG |
| qCvdnaJ-R | TTCCAGGACCAAGTTGCCTAATCG |
| qUBC2D-F | TTCTGAAGGAACTCAAGGAT |
| qUBC2D-R | TGCGTAGGGACTATCTGG |
| qCvHSF30-2-F | AGTTTTATTGTTTGGGACTTGCC |
| qCvHSF30-2-R | ATTAGCGAACTCCCATCTGTC |
| qCvHSP17.3-F | GGAAGGGAACGTGCTGCAGATAAG |
| qCvHSP17.3-R | TGGACTTGACCTCGGGCTTCTTAG |
| qCvHSP17.6-F | AACATGAGGAACGTCCCGATGAAC |
| qCvHSP17.6-R | CCATCAGCAGTGAGTGACGATTTG |
| qCvHSP17.8-F | CAAACACTCGCATCGACTGGAAAG |
| qCvHSP17.8-R | GAAGAACCCTGCCTTCTTCAAC |
| qCvHSP20-F | GGAAACCACCCAATTTGCCAACAC |
| qCvHSP20-R | CTACCTTCTTCAACTTCCACT |
| qCvHSP26.5-F | CTGCTCGTAGTGAAGTTGCTGTAG |
| qCvHSP26.5-R | ATCAAAGGGCGCGACATCCCTATC |
| qCvHSP30.1-F | GATCGTCAAGCCTTCCGCGACTTC |
| qCvHSP30.1-R | CCGTGAAGCTCGTAGCCGTCTTTG |
| qNbHSF30-2-1F | CCAATGGAGGGGCTGCATGAC |
| qNbHSF30-2-1R | TGAGAATCCCAAACAATAAAG |
| qNbHSF30-2-2F | AGCTTTATTGTTTGGGATTC |
| qNbHSF30-2-2R | ATATGTGTTAAGCTGGCGAATG |
| qNbEF1α-F | TGGTGTCCTCAAGCCTGGTAT |
| qNbEF1α-R | ACGCTTGAGATCCTTAACCGC |
